# *Drosophila* Host Model Reveals New *Enterococcus faecalis* Quorum-Sensing Associated Virulence Factors

**DOI:** 10.1371/journal.pone.0064740

**Published:** 2013-05-29

**Authors:** Neuza Teixeira, Sriram Varahan, Matthew J. Gorman, Kelli L. Palmer, Anna Zaidman-Remy, Ryoji Yokohata, Jiro Nakayama, Lynn E. Hancock, António Jacinto, Michael S. Gilmore, Maria de Fátima Silva Lopes

**Affiliations:** 1 ITQB Instituto de Tecnologia Química e Biológica, Universidade Nova de Lisboa, Oeiras, Portugal; 2 Departments of Ophthalmology, and Microbiology and Immunobiology, Harvard Medical School, Boston, Massachusetts, United States of America; 3 CEDOC Faculdade de Ciências Médicas, Universidade Nova de Lisboa, Lisboa, Portugal; 4 Division of Biology, Kansas State University, Manhattan, Kansas, United States of America; 5 Department of Bioscience and Biotechnology, Faculty of Agriculture, Graduate School, Kyushu University, Fukuoka, Japan; 6 IBET Instituto de Biologia Experimental e Tecnológica, Oeiras, Portugal; National Institutes of Health, United States of America

## Abstract

*Enterococcus faecalis* V583 is a vancomycin-resistant clinical isolate which belongs to the hospital-adapted clade, CC2. This strain harbours several factors that have been associated with virulence, including the *fsr* quorum-sensing regulatory system that is known to control the expression of GelE and SprE proteases. To discriminate between genes directly regulated by Fsr, and those indirectly regulated as the result of protease expression or activity, we compared gene expression in isogenic mutants of V583 variously defective in either Fsr quorum sensing or protease expression. Quorum sensing was artificially induced by addition of the quorum signal, GBAP, exogenously in a controlled manner. The Fsr regulon was found to be restricted to five genes, *gelE*, *sprE*, *ef1097*, *ef1351* and *ef1352*. Twelve additional genes were found to be dependent on the presence of GBAP-induced proteases. Induction of GelE and SprE by GBAP via Fsr resulted in accumulation of mRNA encoding *lrgAB*, and this induction was found to be *lytRS* dependent. *Drosophila* infection was used to discern varying levels of toxicity stemming from mutations in the *fsr* quorum regulatory system and the genes that it regulates, highlighting the contribution of LrgAB and bacteriocin EF1097 to infection toxicity. A contribution of SprE to infection toxicity was also detected. This work brought to light new players in *E. faecalis* success as a pathogen and paves the way for future studies on host tolerance mechanisms to infections caused by this important nosocomial pathogen.

## Introduction

*Drosophila melanogaster* is used increasingly as a model for identifying virulence factors of pathogenic microbes, and for elucidating their effects on the host [1]. The fruit fly presents several advantages, such as small size, short life cycle, short generation time, a fully sequenced genome and pre-existing libraries of genetic mutants. In addition, its immune system shares similarities with the mammalian immune system, including genes and pathways. In particular, the *Toll* and *Imd* pathways in *D. melanogaster* have parallels in the mammalian Toll-like (TLR) and interlleukin-1 (IL-1) receptor families, and the mammalian tumour necrosis factor signalling pathway [2]. In 2007, Cox and Gilmore characterized the microbiome of this host and showed that *Enterococcus sp.* and naturally colonize its alimentary canal; and that cytolysin, a toxin expressed by some strains of *Enterococcus faecalis*, contributes to death of the flies when colonized [3]. It is also known that *E. faecalis* are able to kill the flies and induce the *Toll* pathway after infection by septic injury, and that haemocytes (*Drosophila* circulating cells that function as phagocytes) also play a role in flýs defence against these bacteria [4], [5].

Enterococci are Gram-positive bacteria commonly found in gastrointestinal tract consortia, but are also adapted to survive and persist in the environment. In contrast to their benign role as members of the gut flora, select lineages of several enterococcal species have become leading causes of antibiotic resistant nosocomial infection, causing infections of the urinary tract, bloodstream, intra-abdominal and pelvic regions, and surgical sites [6].

*E. faecalis*, the species most frequently associated with nosocomial infections [7], possesses a number of traits that exacerbate the effects of infection. Fsr (*Enterococcus faecalis* sensor regulator) a two-component, quorum sensing regulatory system, was first described in 2000 by Qin *et al.* as a paralog of the Agr system in *Staphylococcus aureus* [7]. Despite similarities, Agr is functionally distinct from Fsr as it uses the RNAIII riboregulator [8]. The *fsr* operon comprises four genes: *fsrA*, *fsrB*, *fsrC* and *fsrD* [9]. The last encodes an auto-inducing cyclic peptide named gelatinase biosynthesis-activating pheromone (GBAP), and this peptide is processed and exported out of the cell by FsrB. Accumulation of GBAP outside cells is sensed by the FsrC histidine kinase, leading to the activation of the response regulator FsrA. Activated FsrA induces expression of the *fsrBDC* genes forming an auto regulatory circuit that results in a rapid, exponential increase in GBAP signalling. Expression of a second operon is induced by FsrA consisting of two cistrons *gelE-sprE*. The first cistron, *gelE*, encodes gelatinase, an extracellular zinc metalloprotease, and the second, *sprE*, encodes a serine protease [7], [10]. Several studies provided evidence that both Fsr and the proteases independently contribute to the pathogenicity of *E. faecalis* in different infection models [11], [12], [13], [14], [15], [16], [17]. The proteases have also been shown to be involved in biofilm formation [18], in translocation across intestinal T84 cells [19], in degradation of antimicrobial peptides (AMPs) from the immune system of *Galleria mellonella* [20], in autolysis regulation [21] and as regulators of Ace surface protein exposure on the surface of *E. faecalis* cells [22], [23].

The exact mechanisms by which Fsr and its regulated proteases contribute to toxicity of infection are not known. This has been confounded in part by unexplained variation in experimental results. In 2005, Singh *et al.* tested *fsrB* and *gelE* mutants in *E. faecalis* strain OG1RF in a rat endocarditis model. Deletion of the proteases led to a greater decrease in endocarditis severity than deletion of *fsrB*. In the absence of *fsrB*, the *gelE* expression was reduced, and the authors postulated that was the reason for the smaller attenuation of *fsrB* mutant [15]. In contrast, studies examining the role of these traits in rabbit endophtalmitis [13], [14], murine and *C. elegans* infection [11], [12], and in a *G. mellonella* infection model [17] all found that *fsrB* deletion led to a greater attenuation than deletion of the proteases. These last results raised the possibility that Fsr could be affecting directly or indirectly more genes or their products than just the proteases. Bourgogne *et al*. compared gene expression in OG1RF with an isogenic *fsrB* deletion mutant, and provided some evidence that Fsr regulates more than *gelE* and *sprE* protease genes [24]. While it is known that host substrates, such as complement components C3, C3a and C5a are targeted by GelE [20], [25], [26], little is known regarding a functional role for SprE in production of host injury and death.

To decipher the role of Fsr-regulated genes in virulence, we used a clonal-complex (CC) 2 strain [27], *E. faecalis* V583, the first vancomycin enterococcal isolate in the US, which was obtained from a chronic bloodstream infection [28]. *E. faecalis* CC2 is the leading multidrug resistant hospital adapted clade [27], [29]. To rigorously characterize the Fsr regulon, we compared gene expression in isogenic mutants in Fsr genes and each of the Fsr-regulated protease genes using microarrays and purified GBAP. *D. melanogaster* was used to examine the individual contribution to virulence of SprE protease and other genes found to be part of the Fsr regulon (or related to it, including EF1097, LrgAB and the two-component system LytRS).

## Results

In order to precisely identify genes for which expression is altered when GBAP reaches effective quorum sensing concentration, we used a *fsrB* mutant, which is unable to produce GBAP, but is able to sense it [30]. We also used single and double protease mutants in the *fsrB* mutant background in order to identify any genes for which expression is indirectly controlled by Fsr through its regulation of protease levels. Table 1 shows key changes in gene expression in V583*ΔfsrB,* V583*ΔfsrBΔgelE*, V583*ΔfsrBΔsprE* and V583*ΔfsrBΔgelEΔsprE* after 10 min of GBAP exposure. Besides genes previously known, or predicted, to be regulated by Fsr through GBAP (*gelE*, *sprE* and *ef1097*) [7], [10], [24], 15 additional genes were differentially regulated by GBAP addition collectively in all four mutants (Table 1). In contrast to previous results using oligo-array study [24], the current approach employed a statistically more robust technology [31] and isolated the effects of only Fsr quorum sensing through the use of mutants and the exogenous quorum molecule.

**Table 1:** Genes differentially expressed upon addition of GBAP to V583*ΔfsrB*, V583*ΔfsrBΔgelE*, V583*ΔfsrBΔsprE* and V583*ΔfsrBΔgelEΔsprE* strains.

Locus	Putative function		Fold Change^1^		
		V583*ΔfsrB*	V583*ΔfsrBΔgelE*	V583*ΔfsrBΔsprEa*	V583*ΔfsrBΔgelEΔsprE*
**EF0411** 3	PTS system mannitol-specific IIBC	−	−	−	−3
**EF0468** 4	LemA family protein	−	+3	−	−
**EF0563** 5	Hypothetical protein	−	−	−	+3
**EF0776** 6	Hypothetical protein	−	+11	−	−
**EF0891** 7	Aspartate aminotransferase putative	−	−	−	−4
**EF0892**	Aminoacid ABC transporter,ATP-binding protein	−	−	−	−3
**EF0893**	Aminoacid ABC transporter/permease		−3	−3	−3
**EF1097**	Putative Bacteriocin	+31	+23	+30	+47
**EF1218** 8	spermidine/putrescine ABC transporter,permease	−	−	−	−3
**EF1351**	Hypothetical protein	−	+6	+8	+4
**EF1352**	Magnesium-translocating, P-type ATPase	+5	+7	+5	+3
**EF1815** 9	Transcriptional regulator, LysR family putative	−	−	+12	+11
**EF1816**	Hypothetical protein, with domain β-lactamase	−	−	+4	+3
**EF1817**	**Serine protease – SprE**	**+60**	**+90**	−	−
**EF1818**	**Gelatinase – GelE**	**+63**	−	**+42**	−
**EF1820**	**Histidine Kinase – FsrC**	**+3**	**+4**	**+3**	**+4**
**EF3193** 2	Antiholin-like protein LrgB	+34	−	−	−
**EF3194** 2	Murein hydrolase regulator LrgA	+79	−	−	−

Fold-change values were obtained by comparing gene expression at 10 min against 0 min post-GBAP addition, by microarray analysis.

1 Fold-change values were obtained by comparing gene expression at 10 min against 0 min post-GBAP addition, by microarray analysis. (+) up-regulated (−) down-regulated;

2 These two genes were up-regulated in the experiments done without GBAP, only in the V583*ΔfsrB* strain with a fold change of +7 for E3193 and +6 for EF3194;

3 *ef0411* is part of the predicted operon *ef0411-0412-0413*, which encodes a mannitol specific PTS-system;

4 LemA-like protein likely involved in cell wall metabolism. LemA proteins contain a predicted amino terminal transmembrane helix and a short extracellular amino terminus. The exact molecular function of this protein is uncertain;

5 Has two predicted transmembrane helixes and a Blast search does not reveal similarity to proteins of known function. Upstream is a putative operon encoding the potassium-transporting ATPase KdpABC (EF0567–EF0569) and the two-component system KdpED (EF0570–EF0571) (TCS12) [62];

6 It has a predicted transmembrane domain at its N-terminus (residues 4 to 20) and the rest of the protein is located outside the cell. It has a predicted thioredoxin fold domain similar to bacteriocin accessory proteins ((http: //www.genome.jp/dbget-bin/www_bget?efa: EF0776);

7 Predicted to facilitate the conversion of aspartate and alpha-ketoglutarate to oxaloacetate and glutamate;

8 Part of the predicted operon *ef1218–ef1224*, which codes for a spermidine/putrescine ABC transporter;

9 EF1815 has 25% amino acid sequence similarity to CidR from *S. aureus* (http: //blast.ncbi.nlm.nih.gov/); EF1816 is a hypothetical protein with a β-lactamase domain, has no transmembrane domain, and is orthologous to PhnP, which is involved in phosphonate metabolism. EF1815 and EF1816 are located upstream of SprE (EF1817), but only EF1816 is located in the positive DNA strand.

### Fsr Dependent Genes

As expected, V583*ΔfsrB* responded to GBAP by substantially increasing the expression of *gelE* (*ef1818*) (fold change 63) and *sprE* (*ef1817*) (fold change 59). To a lesser extent, *fsrC* (*ef1820*) (fold change 3) transcript abundance was also increased. As shown in Table 1, mutation of each protease gene did not affect the expression of the other genes in the *fsr* or *gelE-sprE* operons, showing that the presence of the deletions in these operons did not have polar effects on transcript abundance of the remaining protease gene (V583*ΔfsrBΔgelE* expresses wild type levels of *sprE,* and V583*ΔfsrBΔsprE* expresses wild type levels of *gelE)*. In accordance to previous results by others [24], *fsrA* expression was not affected by GBAP. Genes for which expression was affected by GBAP in all the four mutants are therefore under the direct control of FsrA and not influenced by indirect activities of the proteases on secondary regulators. In addition to Fsr and protease genes, e*f1097* was induced by GBAP addition showing transcript abundance changes (fold change 31) similar to those observed for the protease genes. Transcripts of the *ef1352* gene where more abundant upon GBAP induction, but exhibited an increase of a lower magnitude (fold change 5).

To determine whether a specific promoter motif could be identified upstream of genes found to be regulated by Fsr through its quorum sensing, we compared known [32] and putative promoter regions. The V583 promotor regions of *ef1097*, *gelE* and *fsrB* possess a predicted FsrA binding motif [32]. However, this motif does not occur upstream of *ef1351*. This raises the possibility that induction of *ef1351–ef1352* in our experiments may be related to increased expression of the only gene which was also induced in the four mutants, but not independently controlled, *ef1097*. Alternatively, direct FsrA regulation mechanisms may be more complex than previously suspected.

### Genes Dependent on Simultaneous Fsr and Proteases Activation

Some genes were found to be affected by the presence or absence of proteases, indicating an indirect regulatory pathway. Those only affected if *sprE* was absent (*ef1815, ef1816*); those affected only if either one of the proteases was absent (*ef0893*); those for which mRNA levels were altered only when both proteases were absent (*ef0411*, *ef0563*, *ef0891*, *ef0892*, *ef1218*); those for which mRNA accumulated only in the presence of both proteases (*ef3193* and *ef3194*) and those affected in the absence of only the *gelE* gene (*ef0468*, *ef0776*). These last two genes might respond to the high expression levels of *sprE* in a way yet to be determined. Overall, the twelve genes affected by the combined activation of Fsr and the proteases are putatively involved in different cellular processes, such as regulation, cell-wall metabolism and transport, and some are even of unknown function. Currently available data does not allow us to further clarify the connection between these genes and the Fsr-GelE-SprE system.

### LytRS System is Required for GBAP Induction of *lrgAB* Genes

**Figure 1:**
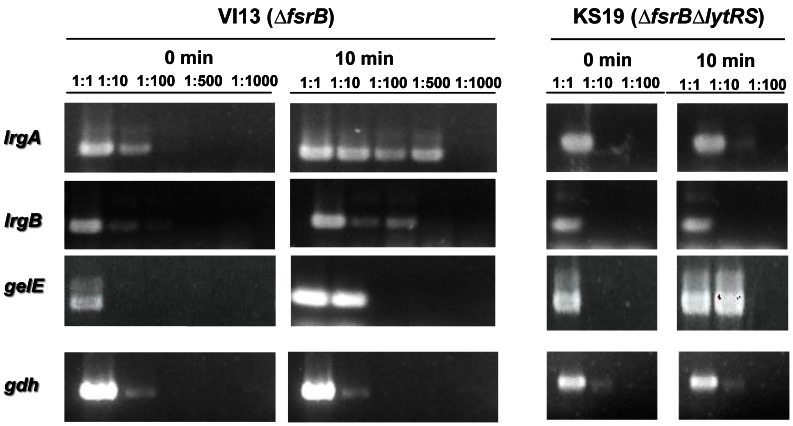
LytRS is required for GBAP induction of *lrgAB* genes. The semi-quantitative RT-PCR shows expression of *lrgAB* genes in the VI13 (Δ*fsrB* mutant) and KS19 (Δ*fsrB*Δ*lytRS* mutant), in the presence of GBAP. Expression of *gelE* and *gdh* were used as positive and negative controls, respectively, of Fsr induction by GBAP and of RNA concentration, respectively. The RNA used for this analysis was previously treated with RNase-free DNase I to remove contaminating DNA.

EF3193–EF3194 correspond to the *lrgAB* genes which, in *S. aureus*, are described to be involved in repression of murein hydrolase activity, decreased autolysis and increased tolerance to penicillin [33]. In *S. aureus* these genes are regulated by the LytRS two-component regulatory system, located immediately upstream of the *lrgAB* genes [34]. There is no data about the function of *lrgAB* genes in *E. faecalis* but it is known that they are also located downstream of *lytRS* homologs, which suggests that in V583 *lrgAB* are regulated by LytRS. In our experiments, *ef3193-3194* mRNA was more abundant upon GBAP induction only in the *fsrB* mutant, suggesting that these genes are not responding directly to FsrA activation, but probably to increased protease GelE and SprE expression, which only occurs when GBAP is added to the *fsrB* mutant. In order to test the hypothesis that the large increase in *lrgAB* abundance was the result of GBAP induction via the LytRS system, we deleted this two-component system from the *fsrB* mutant strain and compared the expression of *lrgAB* genes in the Δ*fsrBΔlytRS* and *fsrB* mutants (Figure 1). We found that GBAP is only able to induce *lrgAB* genes if LytRS is functional. These results were not observed in previous studies of *fsr* regulation in OG1RF [24]. None of the *E. faecalis* Δ*lytRS* or Δ*lrgAB* mutant strains showed different antibiotic resistance profiles (Table S1) nor gelatinase activities when compared to the wild-type strain (data not shown). Low level expression of *lrgAB* genes was observed in the Δ*fsrBΔlytRS* mutant (Figure S1), which points either to a low constitutive expression of those genes or to the existence of another regulator(s) able to modulate their expression.

### Fsr and the Proteases Affect *D. melanogaster* Tolerance to *E. faecalis* Infection

**Figure 2:**
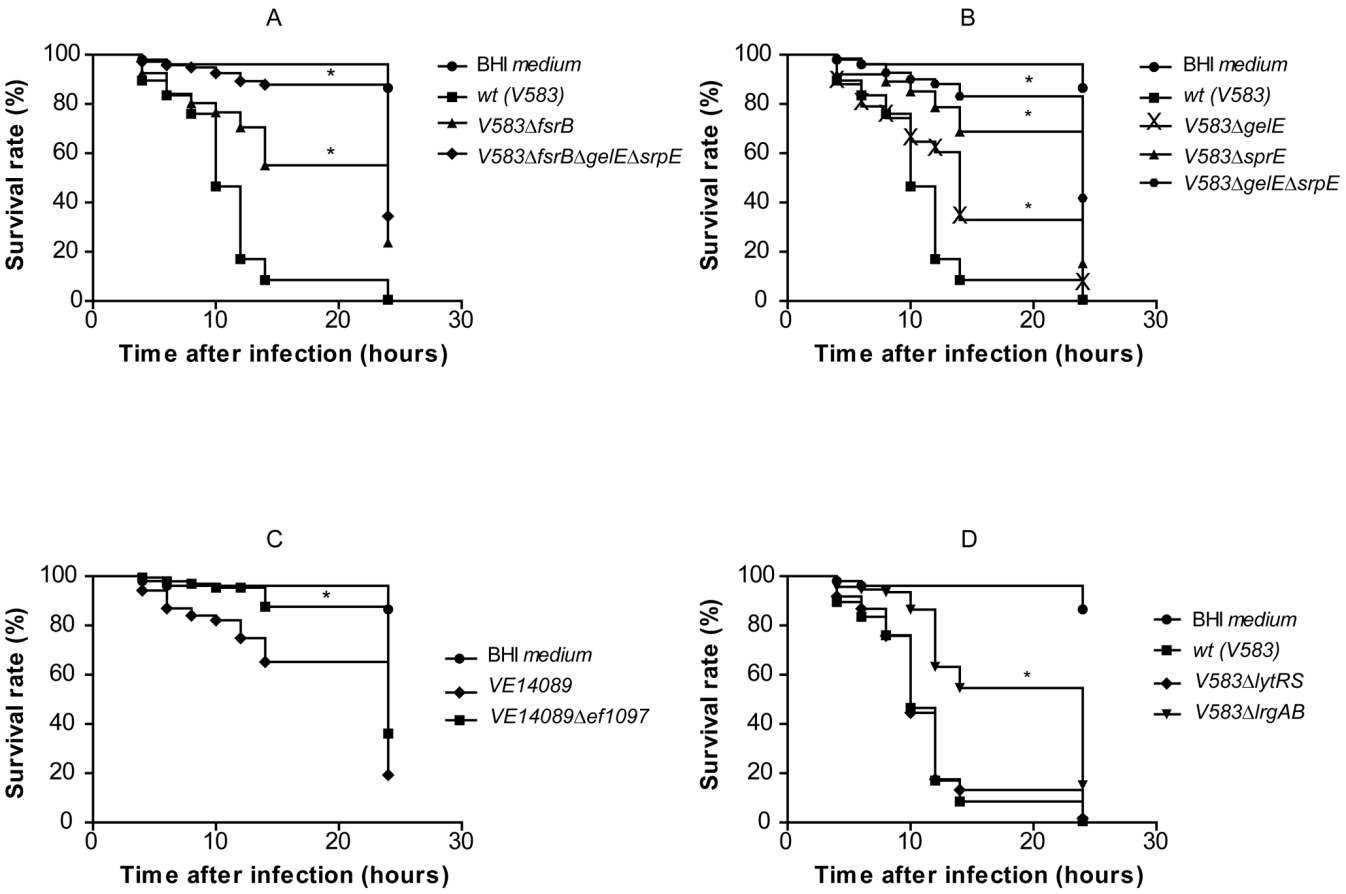
*Drosophila* survival rates upon infection with *E. faecalis* strains. 75 Oregon R (5- to 7-day-old) male adult flies, raised at 25°C, were divided in tubes of 25 flies each, and infected, by septic injury onto the thorax with a thin needle, with V583 (A, B, D) and VE14089 derived strains (C). Data is representative of three independent experiments (225 flies per strain). Curves assigned with an * are significantly different (p<0.0001) from the respective wild-type -infected curve, as determined by log-rank analysis (Table S2).

To test the functional importance of genes found to be directly and indirectly dependent on Fsr, we then tested the virulence of the *fsr*-related mutants in a *D. melanogaster* injection model. We first compared the ability of the triple mutant V583*ΔfsrBΔgelEΔsprE*, to the single V583*ΔfsrB* mutant, and the V583 parental strain, to kill *Drosophila*. The fate of both the host (percentage of survival) and the bacteria (number of CFU) was followed for 24 h. In our assay, 50% of the flies were killed by the wild type strain 10 hours post-injection and after 14 h nearly all flies were dead (Figure 2A). For the same period of infection, the triple mutant V583*ΔfsrBΔgelEΔsprE* strain only killed 15% of the infected flies. 24 h post-injection, the triple mutant V583*ΔfsrBΔgelEΔsprE* was significantly attenuated (see Table S2 for detailed statistical analysis). These results show that the Fsr system and the proteases it regulates contribute measurably to toxicity in this model.

The survival curve of flies infected with the wild type strain shows two different killing rates: until 8 h, V583 strain is able to kill around 3 flies/hour; after this time, and until 12 h, V583 kills flies at a much higher rate, 15 flies/hour. At 8 h post infection, V583 cells reach the cell density considered to be able to induce the activation of the Fsr system in broth culture [9], [35]. Although there is no data on the *in vivo* Fsr expression during *E. faecalis* growth inside the host, we cannot exclude the possibility that the increased killing rate after 8 h is due to induced expression of the proteases.

In order to dissect the contribution of *fsr*-regulated genes to the lethality of infection, we tested these genes separately by infecting the flies with single deletion mutants (Figure 2B). Deletion of both proteases, either in the double protease mutant or in the triple mutant, led to a greater attenuation of virulence then deletion of *fsrB* (p<0.0001, Table S2). Consistent with previous demonstrations that in an *fsrB* mutant strain, proteases are still expressed [15], we observed an attenuation of the virulence in the triple mutant over that of the *fsrB* mutant, suggesting that low level expression of both proteases is enough to induce increased killing of the flies by the *fsrB* mutant. Absence of *gelE* alone produced the lowest attenuation of *E. faecalis* virulence, differing significantly (p<0.0001, Table S2) from the effect of the absence of *sprE* gene alone, which was attenuated to a similar level achieved by deletion of *fsrB* (Table S2). This result points to SprE as having a major role in *E. faecalis* virulence in the *Drosophila* model. All strains grew similarly inside *Drosophila* (Figure S2).

### *ef1097* Contributes to Toxicity in *D. melanogaster* Infection

The large increase in *ef1097* mRNA abundance upon GABP addition, and the fact that it has been previously associated with Fsr system in another *E. faecalis* strain [24], led us to delete this gene to test its role in *E. faecalis* virulence. This mutant was constructed in VE14089, a plasmid cured derivative strain of V583, previously reported in *G. mellonella* to be less virulent than parental V583 strain [36]. Our results confirm that strain VE14089 is less virulent than V583 in the *D. melanogaster* model as well (compare control in Figure 2A and 2C). Previously, we compared the toxicity of V583*ΔfsrBΔgelEΔsprE* and V583*ΔgelEΔsprE* strains in the fly (Figure 2A and 2B). Both strains express *ef1097*, and therefore, the role of this protein was not assessed. Figure 2C clearly shows that deletion of *ef1097* reduces killing of the flies by *E. faecalis*, therefore providing evidence for a role of this bacteriocin in *E. faecalis* toxicity in the fly. As deletion of *ef1097* did not affect the gelatinase production ability of V583 strain (results not shown), the reduction of toxicity does not appear to be due to an effect on expression of *fsr* or the proteases it regulates.

### LrgAB and LytRS Contribute Differently to Death of *D. melanogaster*

LytRS appears to induce *lrgAB* expression upon addition of GBAP to the *fsrB* mutant strain (Figure 1). Interestingly, *lytRS* was previously found to be strongly induced during infection of *G. mellonella,* and proposed to contribute to *E. faecalis* VE14089 virulence in the same model [37]. The importance of LytRS was therefore tested in *Drosophila* infection. Our results (Figure 2D) did not show a significant difference in the fly survival (Table S2) following infection with the *lytRS* mutant as compared to wild type. Our results cannot be compared to those of Hanin *et al*. [37] as both the strains and the infection protocols used were different.

*lgrAB* are still expressed in the *lytRS* mutant. We thus wondered if complete abolishment of its expression would have a more pronounced effect on *D. melanogaster* toxicity than that of its regulator LytRS. The *lrgAB* mutant strain was significantly reduced in toxicity for *D. melanogaster* (Figure 2D, Table S2). This result highlights the relevance of the *lrgAB* operon in infection by *E. faecalis* and constitutes the first report on such a role for this operon in this species.

## Discussion

Assessing the basis for virulence of an opportunistic pathogen, such as *E. faecalis,* is difficult because it is invariably subtle and multifactorial. Research on this topic in recent years has concluded that the sole presence of a gene predicted to induce virulence in a strain does not necessarily imply that the same gene may lead to the same host fate in a different *E. faecalis* strains [17], [38]. Besides the genome background and the host, the manner in which the microbe is introduced also play a roles in determining whether or not a factor contributes to toxicity. *D. melanogaster* has been used as a model host to study pathogenesis because it provides easy handling, fast results, a fully sequenced genome, pre-existing libraries of genetic mutants, the possibility to play on the host side and similarities with the mammal immune system. In this work, we show that it can be used to discern varying levels of toxicity stemming from mutations in the *fsr* quorum regulatory system and the genes that it regulates.

In a representative of the hospital endemic lineage CC2, V583, the Fsr regulon is largely restricted to the five genes, namely *gelE*, *sprE*, *ef1097*, *ef1351* and *ef1352* found to be directly dependent on GBAP-induced Fsr activation, and twelve additional genes found to be dependent on GBAP induction of the proteases. Among these are genes coding for proteins involved in cell-wall, transport and regulatory functions. These genes are thus candidates to link the Fsr-proteases activity with the phenotypes known to be associated to their impairment, namely biofilm formation, adhesion and translocation to/in host-cells, autolysis and host damage and death. This contrasts with previous findings in the more commensal background, OG1RF, which was tested using an X-mer based oligonucleotide array with fewer controls and less redundancy than the Affymetrix microarrays used here. Our experiment assayed the first ten minutes after a burst of GBAP aiming to get clear, measurable and immediate changes in expression, whereas the study by Bourgogne *et al* [24] followed the changes in expression of an *fsrB* mutant spanning different growth stages. Their experimental design likely allowed for further events of differential expression to take place. Whether the differences in results stem from differences in strains, or differences in techniques and experimental approaches used, is not currently known.

In the present study, we found that induction of GelE and SprE by GBAP via the *fsr* regulator resulted in accumulation of mRNA encoding *lrgAB*, and that this induction was *lytRS* dependent, indicating a functional relationship between Fsr and LytRS regulons. In *S. aureus*, autolysis is positively regulated by Agr, a paralog of Fsr, that positively regulates LrgAB [39]. Unlike *S. aureus*, in *E. faecalis* FsrA does not regulate *lrgAB* genes directly, but does so indirectly. Both GelE and SprE have previously been shown to play a role in autolysis regulation in *E. faecalis*, respectively promoting and repressing it [40]. GelE is known to proteolytically activate AtlA [21], a major autolysin. Recently, GelE was also found to control the levels of SalB, a protein with no evident peptidoglycan hydrolytic activity, but affecting the levels of proteins involved in cell-wall synthesis and cell division [41]. A *salB* mutant in OG1-RF strain showed anomalous cell-division and increased autolysis [41]. Given the current knowledge, we could speculate that autolysis regulation could constitute the functional link, found in this study, between Fsr and LytRS. Future studies should address the mechanism behind GelE-SprE regulation of autolytic activities in *E. faecalis* and how they affect the expression of *lrgAB* operon through LytRS regulation.

EF1097 protein, found by Bourgogne *et al.* 2006 [24] to be dependent on Fsr regulation in *E. faecalis* OG1RF, was here confirmed to be true also for the V583 strain. In 2007, Swe *et al.* [42] suggested that *ef1097* gene encodes a precursor of antimicrobial proteins with similarities to the streptococcin SA-M57 in *S. aureus*. EF1097 is conserved in all *E. faecalis* strains (Table S3). Finding this bacteriocin to be similarly regulated in distinct *E. faecalis* strains, namely OG1RF and V583, suggests this is a common feature in the species. QS-activated bacteriocin production may constitute a means to kill surrounding and competing bacteria thus providing competitive advantage to *E. faecalis* when colonizing or infecting a host. The Fsr homologue in *S. aureus*, Agr, is known to regulate the expression of pro-inflammatory peptides, the phenol-soluble modulins (PSM), in a RNAIII independent way [43]. Several roles in pathogenesis have been attributed to these amphipathic peptides [44], including antimicrobial activity [45], biofilm formation, maturation and detachment [46], and cytolytic ability to neutrophils and other human cells [47]. Although the role of EF1097 is not as extensively studied as that of PSMs, their shared features, namely quorum-sensing induction and role in virulence, should direct further studies on EF1097 role in *E. faecalis* biology and interaction with the host.

Despite the inexistence of clues on the EF1097 mechanism of action, bacteriocins have been shown to produce changes in membrane potential and affect transport of magnesium and amino acids [48]. EF1352, which codes for a putative magnesium-translocating P-type ATPase, was induced in all strains used in the microarrays. However, this operon lacks the previously described FsrA binding motif in its promotor region. It is thus licit to speculate that expression of this operon may be dependent on expression of *ef1097*, as this is the only Fsr dependent gene with the FsrA motif not deleted and tested in the microarrays assays. Further studies are needed to understand the link between bacteriocin production and induction of an MgtA transporter, although we could hypothesise that EF1097 could induce ion leakage, which in turn, would induce MgtA.

**Figure 3:**
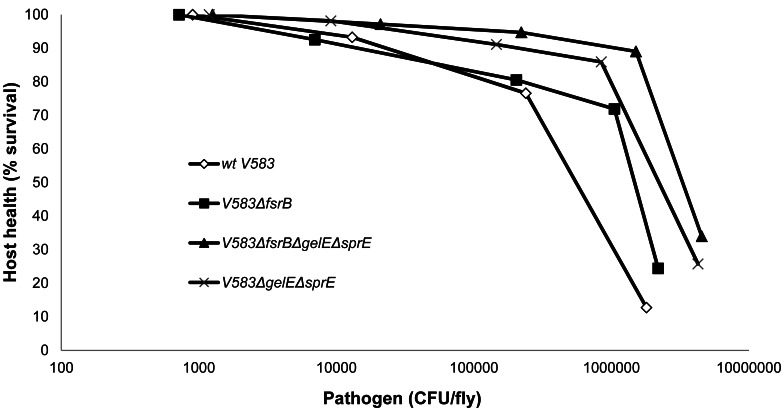
*Drosophila*-health by *E.* *faecalis*-load curve. Source data used to construct this figure was obtained from results on Figure 2, only considering time points at which enough flies alive were available. All strains show two different slopes corresponding to different tolerance values, revealing that at some point (pathogen load value) there is a huge decrease in tolerance to *E. faecalis*. This inflection point corresponds to a lower pathogen load for the wild type strain (10^5^), when compared to the mutant strains (10^6^). For 10^6^ value of pathogen load, the wild type induced only 10% survival in the *Drosophila* population, as opposed to 90% survival of the *Drosophila* population infected with the triple mutant.

Despite different mortality curves were produced upon infection of *Drosophila* with the tested mutants, they all grew similarly inside the host. Hosts have two ways to deal with an infection: resistance and tolerance [36], [37]. Resistance is related with pathogen load and with mechanisms used to kill the pathogens: more resistant hosts have fewer pathogens. Tolerance is a consequence of the host ability to overcome the fitness cost imposed upon infection and induction of the immune system and is related to the ability of the host to remain healthy. Tolerance can be defined and measured from the slope of the health-by microbe curve. We plotted the flýs survival against pathogen load, assuming host population survival as a measure of its health (Figure 3), and confirmed that inactivation of Fsr and the two proteases increased flýs tolerance to *E. faecalis*, whereas flies showed similar resistance towards all studied *E. faecalis* strains. Mechanisms involved both in tolerance [49], [50] and resistance [5], [51], [52]of *Drosophila* towards enterococcal infections have been identified. If we understand how the *E. faecalis* virulence factors studied in this work affect the flýs tolerance mechanisms and responses, we can postulate that future approaches to fight enterococci can be through improving host tolerance, providing an alternative, or complementary, approach to bacterial killing by use of antibiotics.

GelE is known to be able to degrade several host proteins. Therefore, besides its ability to degrade host immune factors, this protease may be involved in host tissue injury. Recently, GelE has also been implicated in release of Ace protein from the surface of *E. faecalis* cells in OG1RF strain [22]. In that study, authors showed that deletion of *gelE* gene increased the number of Ace proteins bound to the surface of the bacterial cells, increasing adherence to collagen. In the insect model *G. mellonella*, collagen adherence has been shown to be required for invasion and virulence [53]. Although this remains to be proven true for *Drosophila*, it is licit to speculate that the lower attenuation of the *gelE* mutant in this insect host model could be due to increased adherence to host cells and proteins. Despite considered to be cell-bound, SprE is also able to degrade host proteins, such as insulin and fibrinogen, but not immune system elements, such as complement from human serum or Cecropin from insect hemolymph [20]. Its major contribution to host death proven in this work needs thus urgent clarification.

**Figure 4:**
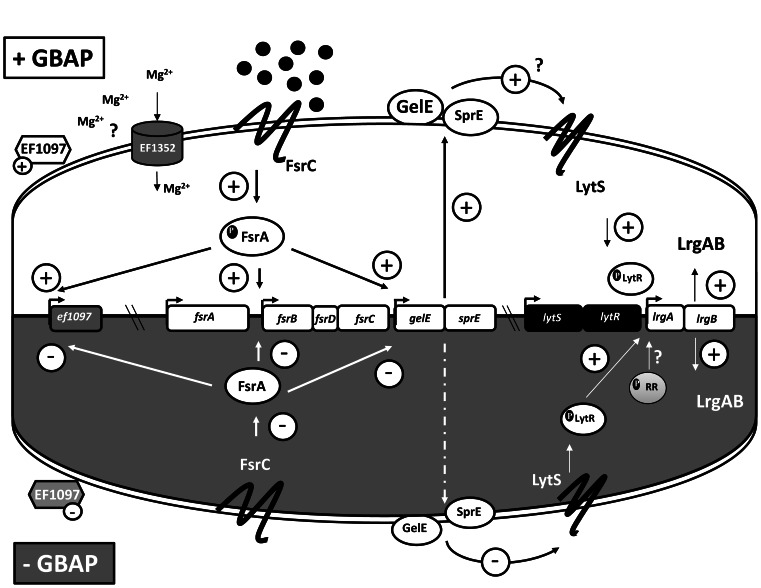
GBAP-dependent regulatory network. Once the GBAP (black disks) concentration outside cells reaches a certain threshold (upper part of the cell), the Fsr system is activated, and the FsrA regulator induces expression of *gelE*, *sprE* and *ef1097* genes. Both produce proteins which will be located to the cell membrane and cell wall. Although GelE is loosely bound to the cell, it will also be released from it. The induced expression of *ef1352*, which encodes a putative MgtA protein, by GBAP is likely due to increased amounts of EF1097, predicted to be a bacteriocin. EF1352 could function as an auto-immunity factor against EF1097. The increased level of GelE and SprE proteins in the cell-wall in response to GBAP are proposed to induce changes sensed by LytS protein, which in turn, activates LytR, responsible for induction of *lrgAB* genes. When no GBAP is produced (lower part of the cell) *ef1097* is not expressed, but both GelE and SprE are still produced, although in lower amounts (dotted line). In this situation, *lrgAB* genes are still expressed, but the increment in their expression during growth in the exponential phase (assayed during microarrays performed without GBAP) is not due to the QS molecule. As we found that *lrgAB* can still be expressed in a *lytRS* mutant, we propose that this is not the only regulator able to induce expression of that operon.

This work brought to light new players (Figure 4) in Fsr role in *E. faecalis,* namely LrgAB operon, which will help unravel the bacterial programmed cell death which, in turn, may help discover new approaches to control this important nosocomial pathogen. Moreover, *Drosophila* was successfully established as a model to study virulence associated genes in *E. faecalis*, highlighting LrgAB and EF1097 as novel virulence factors induced by QS. Using *Drosophila* as a model also allowed us to show that SprE is, *per se*, a relevant player in host injury and to suggest that *E. faecalis* success during septic injury is not due to GelE acting as a bacterial defence against the flies AMPs, but that it could rather be through host injury.

## Materials and Methods

### Bacterial Strains and Plasmids

Strains and plasmids used in this study are listed in Table 2. *E. faecalis* strains were grown either in BHI, M17 broth/agar (Oxoid) or Enterococcel Agar (Quilaban) at 37°C, unless a different growth temperature is specified. *Escherichia coli* strains were grown in LB medium (Sigma) at 37°C with agitation. The following antibiotic concentrations were used: with *E. faecalis*, tetracycline 30 µg/ml; with *E. coli*, ampicillin 150 µg/ml and tetracycline 150 µg/ml.

**Table 2:** Strains, plasmids and primers used in this study.

Strains	Relevant characteristics	Reference
***E. coli***		
*DH5α*	F^−^ *Ø*80d*lacZ*ΔM15 Δ(*lacZYA*-*argF*)U169 *deoR recA1 endA1 hsdR17*(r_K_^−^ m_K_^−^ ) *phoA supE44* λ^−^ *thi*-1 *gyrA96 relA1*	[63]
*TG1 RepA*	*supE hsdD5 th*i (Δ*lac-proAB*) F^−^ (*traD36 proAB-lacZΔ*M15) repA	[64]
VE14188	GM1674 (dam− dcm− repA+)	[36]
***E. faecalis***		
V583	Clinical isolate, TIGR sequence strain; Vn^R^	[28]
VE14089	V583 free of replicating plasmids	[36]
VI13	*E. faecalis* V583Δ*fsrB*, GelE−, SprE−, GBAP−	[30]
MG01	*E. faecalis* V583Δ*fsrBΔgelE*; GelE−, SprE−, GBAP−	This study
MG02	*E. faecalis* V583Δ*fsrBΔsprE*; GelE−,SprE−, GBAP−	This Study
MG03	*E. faecalis* V583Δ*fsrBΔgelE*Δ*sprE*; GelE−, SprE−, GBAP−	This Study
VT01	*E. faecalis* V583Δ*gelE,* GelE−, GBAP+	[40]
VT02	*E. faecalis* V583Δ*sprE*, SprE−, GBAP+	[40]
VT03	*E. faecalis* V583Δ*gelEΔsprE*, GelE−, SprE−, GBAP+	[40]
KS17	*E. faecalis* V583Δ*lytRS,* GelE+, SprE+, GBAP+	This study
KS18	*E. faecalis* V583Δ*lrgAB,* GelE+, SprE+, GBAP+	This study
KS19	*E. faecalis* V583Δ*fsrB*Δ*lytRS,* GelE−, SprE−, GBAP−	This study
SAVE38	*E. faecalis* VE14089Δ*ef1097,* GelE+, SprE+, GBAP+	This study
***Plasmids***		
*pGEM-T*	High copy plasmid, Amp^R^	Promega
*pG+host9*	*E. faecalis* thermosensitive plasmid, Ery^R^	[59]
pLT06	Temperature-sensitive cloning vector, Cm^R^	[65]
pVI02	pLT06 containing engineered *fsrB* deletion	[30]
pVT01	pLT06 containing engineered *gelE* deletion	[40]
pVT02	pLT06 containing engineered *sprE* deletion	[40]
pVT03	pLT06 containing engineered *gelEsprE* deletion	[40]
pKS103	pLT06 containing engineered *lytSR* deletion	This study
pKS104	pLT06 containing engineered *lrgAB* deletion	This study
*pSAVE37*	pGEM-T containing engineered EF1097 deletion	This study
*pSAVE38*	pG+host9 containing engineered EF1097 deletion	This study
***Primers***		
EF1097_1	AAG ACA ACA CGGGATAACACTCG	This study
EF1097_2	GCTTAGCCCACATTGAACTGCTGTCATTAGTAATGCCATCGCC	This study
EF1097_3	GCAGTTCAATGTGGGCTAAGC	This study
EF1097_4	CTGAGTTACGGTCCATCCTTCTTCC	This study
LytP1	GAGAGAATTCGCTTGGGAACTTCATTGC	This study
LytP2	CTCTGGATCCGACCACACCGGCACCTCC	This study
LytP3	GAGAGGATCCGTTAGCCGTTCATACGTC	This study
LytP4	CTCTCTGCAGGGTACGGCAATCGCTGTTG	This stud
LytUp	GTATCAACGGTATGAATACGG	This study
LytDown	AATGCAATTCGACCCAAGGC	This study
LrgP1	GAGAGAATTCGGAAAGACGACAGTGACTTC	This study
LrgP2	CTCTGGATCCTTCCATTCTTCTTCGCTCCCT	This study
LrgP3	GAGAGGATCCGCAACGGTCATTGGTCTATAA	This study
LrgP4	CTCTCTGCAGGCCTGCGAATAACTGGTTGA	This study
LrgUp	CCATCAAGCATGCATTTGGC	This study
LrgDown	TGGTACCGCTTGTTTTGACG	This study
mgelE_2	AAC GGA TAA CAC AGG GG	[17]
gelE	TCA TTC ATT GAC CAG	[17]
lrgA_fw	GGGCTTGTTCATTTCCCC	This study
lrgA_rv	AAGGCGCCCGTCCAACCAG	This study
lrgB	TTCTATGCCAACTGCCACAC	This study
mlrgB	AAGGTTTCTTCTTATTTACGCC	This study
gls24_f	TGCGTGGTAGAATACGGCAAAG	This study
gls24_rv	GTCCATATGTCGCATGTTGC	This study

### Antibiotic Resistance Assay

Resistance to different antibiotics (Ciprofloxacin, Penicillin, Sulphamethoxazole, Vancomycin, Nitrofurantoin, Ofloxacin, Ampicillin, and Ceftriaxone) was determined according to the recommendations of the disk providers (Oxoid) [54], and results were interpreted according to the recommendations of the Clinical and Laboratory Standards Institute (CLSI, formerly NCCLS) (http: //www.clsi.org/).

### General DNA Techniques

General molecular biology techniques were performed by standard methods. Restriction enzymes, polymerases and T4 DNA ligase were used according to manufacturers’ instructions. PCR amplification was performed using a Biometra thermocycler. When necessary, PCR products and DNA restriction fragments were purified with purification kits (Macherey-Nagel). Plasmids were purified using the Miniprep kit (Macherey-Nagel). Electrotransformation of *E. coli* and *E. faecalis* was carried out as described by Dower *et al*. (1988) and Dunny *et al.* (1991), using a Gene Pulser apparatus (Bio-Rad) [55], [56]. Plasmid inserts and mutant sequence were confirmed by sequencing at StabVida (Portugal).

### Mutant Construction

*E. faecalis* V583 mutants (MG01[V583*ΔfsrB*Δ*gelE*]; MG02[V583*ΔfsrB*Δ*sprE*]; and MG03[V583*ΔfsrBΔgelE*Δ*sprE*] were constructed by introducing pVT01(*ΔgelE*), pVT02(*ΔsprE*), and pVT03(*ΔgelEΔsprE*), respectively into the VI13[V583Δ*fsrB*] strain and selecting for protease gene deletions essentially as described by Thomas *et al*. 2009 [21]. These strains are still responsive to external GBAP, but are not able to produce the QS molecule, as is the case of VI13[V583*ΔfsrB*] [30]. Construction of KS17[V583*ΔlytSR*] and KS18[V583*ΔlrgAB*] mutants was done similarly to the method described by Thurlow *et al*. using the marker less deletion vector pLT06 [57]. In brief, flanking regions of *lytSR* and *lrgAB* were amplified from *E. faecalis* V583 chromosomal DNA by PCR with primers LytP1, LytP2, LytP3, LytP4 and LrgP1, LrgP2, LrgP3, LrgP4 respectively (Table 2). The flanking PCR fragments were ligated together following BamHI digestion and reamplified by PCR using the external primers P1 and P4, for both the *lytSR* and *lrgAB* deletion constructs. The resulting amplicons were digested with EcoRI and PstI and cloned into similarly digested pLT06 to create pKS103 (Δ*lytSR*) and pKS104 (Δ*lrgAB*). The resulting plasmids were confirmed by restriction analysis and sequenced. Plasmids were introduced into *E. faecalis* V583 by electroporation and selection of the desired mutant was performed as described [57]. To create KS19[V583Δ*fsrB*Δ*lytSR*], VI13 was transformed with pKS103 (Δ*lytSR*) and selection for deletion of *lytSR* was performed as described [57].

*E. faecalis* V583Δ*ef1097* was constructed essentially as described by Brinster *et al*. (2007) [58] in strain VE14089 [36]. Briefly, flanking regions of EF1097 were amplified from chromosomal DNA of V583 by PCR with primers EF1097_1, EF1097_2, EF1097_3 and EF1097_4 respectively (Table 2). The two cognate PCR fragments were fused by PCR using the external primers EF1097_1 and EF1097_4 for EF1097, respectively, and the resulting product was cloned into pGEM-T (Promega). The inserted PCR fragment was removed from its cloning vector by restriction enzymes and subsequently cloned into pG+host9 plasmid [59], which was then electroporated into *E. faecalis* VE14089. The *ef1097* single- and double crossover mutants were selected as described by Brinster *et al.* (2007) [58], [59]. Successful targeted mutations of *ef1097* were first identified by PCR screening and were confirmed by sequencing (StabVida, Portugal), and analysed by Vector NTI program (Invitrogen).

### RNA Extraction and cDNA Synthesis for Microarrays

*E. faecalis* strains were grown in BHI, at 37°C, until 0.4 OD (600 nm). At this point, purified GBAP, prepared as previously described [29], was added to a final concentration of 10 nM in the culture. This concentration was previously shown to be able to induce the Fsr system [9], [36]. In order to determine the effect of GBAP induction at a time in growth when we knew, from previous work [35], that the Fsr system was not yet fully activated, we chose 0.4 OD to add GBAP. The quorum-sensing molecule was added to induce the Fsr quorum-sensing system in strains which lack the ability to produce the GBAP molecule, but are still able to sense it. At time zero (immediately after GBAP addition) and after 10 min post-GBAP addition, RNA was extracted from cells and used to synthesize cDNA and perform microarray transcriptional analysis. Experiments without GBAP were also performed. To prepare samples for Affymetrix GeneChip analysis, a previously published protocol was used with few modifications [60]. Briefly, RNA was stabilized with RNA protect (Qiagen) and RNA was isolated with RNeasy columns per the manufacturer's instructions (Qiagen). Samples were treated with RNase-free DNase I (Roche) to remove contaminating DNA, and the absence of contaminating DNA was confirmed by PCR. RNA integrity was verified using agarose gel electrophoresis of glyoxylated samples (Ambion). cDNA was prepared from RNA using Superscript II Reverse Transcriptase (Invitrogen) with random (N_6_) priming. cDNA was fragmented with dilute DNase I (Roche) and fragments were biotinylated with the BioArray Terminal Labeling Kit (Enzo Life Sciences) prior to hybridization.

### Affymetrix GeneChip Analysis

Samples were hybridized to a previously described custom *E. faecalis* Affymetrix GeneChip [27] and scanned at the University of Iowa DNA Core Facility. All microarray experiments were performed in duplicate. Data was analysed using Affymetrix GeneChip Operating Software, which identifies probe sets with statistically significant hybridization over background (i.e. presence versus absence calls) and among those, identifies probe sets for which hybridization is significantly increased or decreased in pairwise comparisons of microarray experiments. Signal log ratios for differentially expressed probe sets were averaged and converted to fold change values. Only genes with ≥3-fold differential expression were considered. The data discussed in this publication have been deposited in NCBI's Gene Expression Omnibus [61] and are accessible through GEO Series accession number GSE42036 (http: //www.ncbi.nlm.nih.gov/geo/query/acc.cgi?acc=GSE42036).

### Semiquantitative RT-PCR

RNA was extracted from strains V583Δ*lytRS* and V583Δ*fsrB* grown in BHI broth at 37°C. Briefly, overnight cultured cells were diluted 1∶100 and growth was monitored by following OD600. Cells were collected in the same conditions as those used for RNA extraction for microarrays. Total RNA was extracted and purified with an RNeasy Mini kit (Qiagen). RNA integrity was checked by electrophoresis on a 1% agarose gel (RNase free). cDNA was synthesized using random primers (Roche Diagnostics), 3 mg total RNA and a Transcriptor High Fidelity cDNA Synthesis kit (Roche Diagnostics). Serial dilutions of V583Δ*lytRS* and V583Δ*fsrB* cDNA were used for PCR in order to amplify cDNA of *lrgA* (primers: lrgA, mlrgA), *lrgB* (primers: lrgB, mlrgB) and *gelE* (primers: mgel_2, gelE) (Table 2).

### *D. melanogaster* Infection

Oregon R male flies were injected with 50 nl of bacteria at 0.02 OD (600 nm) from one of the strains: V583, V583*ΔfsrBΔgelE*, V583*ΔfsrBΔsprE*, V583*ΔfsrBΔgelEΔsprE*, V583*ΔlytRS*, V583*ΔlrgAB*, VE14089 and VE14089*Δef1097*. As control, flies were injected with the same volume of BHI medium. Male flies were anesthetized with CO_2_ and the injections were carried out with a pulled glass capillary needle using a nano-injector (Nanoliter 2000, World Precision Instruments). Reproducibility was measured by determining the number of bacteria injected at time zero. Injected flies were placed at 29°C, 65% humidity. Seventy-five flies were assayed for each survival curve, and they were placed in three vials of 25 flies each. Each experiment was repeated three times, making a total of 225 flies tested per strain in each set of three replicates, to ensure high confidence results. Death was recorded at 0, 4, 6, 8, 10, 12, 14 and 24 h hours post-injection. All experiments were performed at least three times. Following challenge with bacteria, six individual flies were collected (at 0 h, 4 h, 8 h, 12 h and 24 h), homogenized, diluted serially, and plated onto Enterococcel agar (Quilaban). *E. faecalis* CFUs (colony forming units) were determined by testing three groups of six flies for each time point.

### Percentage of Similarities between V583 Genome and Other Genomes Published

The percentage of similarities was made with blast program (http: //blast.ncbi.nlm.nih.gov/). The genomes that were used on this analysis were from Broad Institute page (http: //www.broadinstitute.org/annotation/genome/enterococcus_faecalis/MultiHome.html) and compared with V583 genome (http: //www.ncbi.nlm.nih.gov/nuccore/NC_004668.1).

### Statistical Analysis

Statistical analysis of *Drosophila* survival was performed using GraphPad Prism software version 5.03. Survival curves were compared using Log-rank and Gehan-Breslow-Wilcoxon tests. Statistical analysis of *Drosophila* survival was performed using t-test.

## Supporting Information

Figure S1 ***lrgAB*** **expression in the absence of GBAP.** The semi-quantitative RT-PCR shows expression of *lrgAB* genes in the VI13 (Δ*fsrB* mutant) and KS19 (Δ*fsrB*Δ*lytRS* mutant) strains, in the absence of GBAP. Expression of *gelE* and *gdh* were used as negative and positive controls, respectively. The RNA used for this analysis was previously treated with RNase-free DNase I to remove contaminating DNA and PCR was done in order to confirm absence of DNA from the RNA samples analysed.
(TIF)

Figure S2 ***E. faecalis*** **growth curves in injected flies.** Oregon R (5- to 7-day-old) male adult flies, raised at 25°C, were divided in tubes of 25 flies each, and infected, by septic injury onto the thorax with a thin needle, with V583 mutants. Flies were collected at 0, 4, 8, 12, and 24 h. Three groups of six flies for each time point were homogenized and plated in Enterococcel agar and *E. faecalis* CFUs were determined.
(TIFF)

Table S1 (DOC)

Table S2 (DOC)

Table S3 (DOC)
