# Supplementary material for: Drosophila Host Model Reveals New Enterococcus faecalis Quorum-Sensing Associated Virulence Factors
Source: PLoS One. 2013 May 29;8(5):e64740. doi: 10.1371/journal.pone.0064740 (PMC3667150; doi:10.1371/journal.pone.0064740)
Supplement: Table S1 — (DOC) [file pone.0064740.s003.doc]

Table S1 – Antibiotic resistance profiles of *E. faecalis* V583 derivative mutants. Values correspond to diameter of halos (µm) around antibiotic disks.

| **Strain** | **Ciprofloxacin** | **Penicillin** | **Sulphamethoxazole** | **Vancomycin** | **Nitrofuratoin** | **Ofloxacin** | **Ampicilin** | **Ceftriaxone** |
| --- | --- | --- | --- | --- | --- | --- | --- | --- |
| **V583** | 24,03 | 22,02 | 30,09 | 10,06 | 22,09 | 20,08 | 28,05 | 15,02 |
| **V583*∆lrgAB*** | 24,01 | 21,08 | 29,06 | 10,06 | 23,03 | 20,08 | 28,09 | 16,04 |
| **V583*∆lytRS*** | 23.03 | 21,03 | 30,08 | 10,02 | 21,07 | 20,08 | 27,04 | 11,09 |
| **V583*∆fsrB*** | 24,09 | 21,03 | 30,00 | 10,04 | 20,06 | 20,01 | 28,04 | 15,0 |
