# Supplementary material for: Drosophila Host Model Reveals New Enterococcus faecalis Quorum-Sensing Associated Virulence Factors
Source: PLoS One. 2013 May 29;8(5):e64740. doi: 10.1371/journal.pone.0064740 (PMC3667150; doi:10.1371/journal.pone.0064740)
Supplement: Table S2 — (DOC) [file pone.0064740.s004.doc]

Table S2 – Statistical analysis of Figure 2 using Kaplan-Myer curves. Experimental and control populations are compared using Log-Rank and Wilcoxon tests (ChiSquare and p-values). Analysis was performed using GraphPad Prism statistical software.

| **Strain 1** | **Strain 2** | **ChiSquare**  Log Rank Wilcoxon | | **p-value**  Log Rank Wilcoxon | |
| --- | --- | --- | --- | --- | --- |
| ***V583wt*** | *V583∆fsrB* | 112,5 | 83,09 | <0,0001 | <0,0001 |
|  | *V583∆fsrB∆gelE∆sprE* | 272,0 | 237,9 | <0,0001 | <0,0001 |
|  | *V583∆gelE* | 41.32 | 25.85 | <0,0001 | <0,0001 |
|  | *V583∆sprE* | 162.9 | 136.1 | <0,0001 | <0,0001 |
|  | *V583∆gelE∆sprE* | 286.4 | 252.0 | <0,0001 | <0,0001 |
|  | *V583∆lytRS*  *V583∆lrgAB* | 0.389  120.1 | 0.013  107.7 | ns  <0,0001 | ns  <0,0001 |
| ***V583∆fsrB*** | *V583∆gelE*  *V583∆sprE*  *V583∆gelE∆sprE*  *V583∆fsrB∆gelE∆sprE* | 24.38  0,2742  36.26  28.73 | 19.12  1.163  39.45  32.80 | <0,0001  ns  <0,0001  <0,0001 | <0,0001  ns  <0,0001  <0,0001 |
| ***V583∆gelE*** | *V583∆sprE*  *V583∆gelE∆sprE*  *V583∆fsrB∆gelE∆sprE* | 40.56  128.5  120.1 | 39.85  126.5  119.5 | <0,0001  <0,0001  <0,0001 | <0,0001  <0,0001  <0,0001 |
| ***V583∆sprE*** | *V583∆gelE∆sprE*  *V583∆fsrB∆gelE∆sprE* | 38.30  31.46 | 36.28  31.10 | <0,0001  <0,0001 | <0,0001  <0,0001 |
| ***V583∆gelE∆sprE*** | *V583∆fsrB∆gelE∆sprE* | 0,6964 | 0,5534 | ns | ns |
| ***V583∆lytRS*** | *V583∆lrgAB* | 95.58 | 95.15 | <0,0001 | <0,0001 |
| ***V583∆ABC*** | *V583∆ABC∆ef1097* | 30.00 | 32.61 | <0,0001 | <0,0001 |
