# Supplementary material for: Drosophila Host Model Reveals New Enterococcus faecalis Quorum-Sensing Associated Virulence Factors
Source: PLoS One. 2013 May 29;8(5):e64740. doi: 10.1371/journal.pone.0064740 (PMC3667150; doi:10.1371/journal.pone.0064740)
Supplement: Table S3 — (DOC) [file pone.0064740.s005.doc]

Table S3 – Similarity (%) between *E. faecalis* V583 proteins (found to have their genes differentially expressed in the microarrays experiments) and sequenced genomes from Pubmed and *Enterococcus* group Sequencing Project, Broad Institute of Harvard and MIT (http://www.broadinstitute.org/). Grey shade represents absence of gene coding for the protein.

|  | Fsr-dependent proteins | | | | | | | | Fsr proteins | | | | |
| --- | --- | --- | --- | --- | --- | --- | --- | --- | --- | --- | --- | --- | --- |
| **Genomes** | **EF0411** | **EF0412** | **EF0413** | **EF1097** | **EF1351** | **EF1352** | **EF3193** | **EF3194** | **EF1817** | **EF1818** | **EF1820** | **EF1821** | **EF1822** |
| ***E. faecalis*** |  |  |  |  |  |  |  |  |  |  |  |  |  |
| OG1RF | 99 | 99 | 99 | 99 | 99 | 99 | 99 | 99 | 98 | 99 | 99 | 99 | 99 |
| dg1 | 98 | 98 | 97 | 98 | 98 | 99 | 98 | 100 | 98 | 98 | 98 | 97 | 99 |
| atcc_4200 | 98 | 98 | 97 | 98 | 98 | 99 | 98 | 99 | 98 | 98 | 98 |  |  |
| ch188_1 | 98 | 98 | 97 | 98 | 100 | 99 | 99 | 99 | 98 | 98 | 98 | 98 | 98 |
| d6_1 | 98 | 98 | 96 | 98 | 100 | 99 | 98 | 99 |  |  |  |  |  |
| ds5 | 98 | 98 | 98 | 98 | 100 | 99 | 98 | 99 |  |  |  |  |  |
| e1sol_1 | 98 | 98 | 97 | 97 | 97 | 99 | 97 | 99 | 98 | 98 | 98 | 97 | 99 |
| fly1_1 | 98 | 98 | 97 | 97 | 97 | 99 | 98 | 99 | 98 | 97 | 98 | 97 | 99 |
| hip11704_1 | 98 | 98 | 98 | 98 | 100 | 99 | 98 | 99 | 98 | 98 | 98 |  |  |
| jh1_1 | 98 | 98 | 98 | 98 | 100 | 99 | 98 | 99 | 98 | 97 | 98 | 97 | 98 |
| merz96_1 | 98 | 98 | 97 | 97 | 98 | 99 | 98 | 98 | 100 | 99 | 98 | 97 | 100 |
| t11_1 | 99 | 97 | 98 | 98 | 100 | 100 | 98 | 99 | 99 | 98 | 98 | 97 | 99 |
| t1_1 | 98 | 98 | 98 | 98 | 98 | 99 | 98 | 99 | 98 | 98 | 98 | 97 | 99 |
| t2_1 | 98 | 97 | 98 | 98 | 100 | 99 | 98 | 98 | 98 | 98 | 98 |  |  |
| t3_1 | 98 | 98 | 98 | 98 | 100 | 99 | 98 | 99 | 99 | 98 | 98 | 97 | 99 |
| t8_1 | 98 | 98 | 98 | 98 | 97 | 99 | 98 | 99 | 98 | 98 | 98 |  |  |
| x98_1 | 98 | 98 | 98 | 98 | 98 | 99 | 98 | 99 | 98 | 98 | 98 | 97 | 98 |
| 62 | 99 | 99 | 99 | 99 | 100 | 99 | 99 | 99 | 99 | 99 | 99 |  |  |
| ***E. faecium*** |  |  |  |  |  |  |  |  |  |  |  |  |  |
| 1,141,733_1 | 80 | 81 |  |  |  |  |  |  |  |  |  |  |  |
| 1,230,933_1 | 79 | 82 | 81 |  |  |  |  |  |  |  |  |  |  |
| 1,231,408_1 | 79 | 82 | 81 |  |  |  |  |  |  |  |  |  |  |
| 1,231,410_1 | 79 | 82 | 81 |  |  |  |  |  |  |  |  |  |  |
| 1,231,501_1 | 79 | 82 | 79 |  |  |  |  |  |  |  |  |  |  |
| 1,231,502_1 | 79 | 82 | 81 |  |  |  |  |  |  |  |  |  |  |
| com12_1 | 80 | 81 |  |  |  |  |  |  |  |  |  |  |  |
| com15 | 79 | 81 |  |  |  |  |  |  |  |  |  |  |  |
| E. gallinarum  _eg2_1 | 80 | 79 | 81 |  |  | 78 |  |  |  |  |  |  |  |
| E. Casseliflavus  _ec30_1 | 80 | 83 | 86 |  |  | 80 |  |  |  |  |  |  |  |
